# Hypertonic stress modulates eNOS function through O-GlcNAc modification at Thr-866

**DOI:** 10.1038/s41598-021-90321-4

**Published:** 2021-05-28

**Authors:** Chang Li, An He, Yongzheng Guo, Xiyang Yang, Minghao Luo, Zhe Cheng, Longxiang Huang, Yong Xia, Suxin Luo

**Affiliations:** 1 Division of Cardiology, The First Affiliated Hospital of Chongqing Medical University, Chongqing, 400016 China; 2 Institute of Life Science, Chongqing Medical University, Chongqing, 400016 China; 3 Division of Cardiovascular Medicine, Department of Molecular and Cellular Biochemistry, Davis Heart and Lung Research Institute, The Ohio State University College of Medicine, 473 West 12th Avenue, Columbus, OH 43210 USA

**Keywords:** Biochemistry, Cell biology, Cardiology

## Abstract

O-GlcNAcylation, an energy-sensitive posttranslational modification, can regulate the activity of endothelial nitric oxide synthase (eNOS). Previous studies found that Thr866 is the key site for low-glucose-mediated regulation of eNOS O-GlcNAc. However, it is not known whether this activity functions through the Thr866 site concomitant with other physical and chemical factors. Therefore, we first explored the effects of physical and chemical factors on eNOS O-GlcNAc and its Thr866 site. In this study, hypertonic stress, hyperthermia and hydrogen peroxide all increased the expression levels of eNOS O-GlcNAc, whereas hypoxia and high levels of alcohol had no effect. on the expression levels of eNOS O-GlcNAc; by contrast, low pH led to a decrease in eNOS O-GlcNAc levels. Notably, eNOS O-GlcNAc protein levels were unchanged after Thr866 site mutation only under hypertonic conditions, suggesting that hypertonic stress may act through the Thr866 site. Upon exploring the mechanism of hypertonic stress on eNOS O-GlcNAc activity and function, we found that hypertonic stress can upregulate the expression of O-linked N-acetylglucosamine (GlcNAc) transferase (OGT), which is dependent on AMPK. When AMPK was knocked out, the upregulation of OGT expression and increased O-GlcNAc modifications induced by hypertonic stress were reversed.

## Introduction

Endothelial nitric oxide synthase (eNOS) is necessary for the production of NO, which can regulate the diastolic and systolic functions of the cardiovascular system^1^. Initially, studies^1,2^ reported that eNOS activity is triggered by an increase in intracellular concentrations of free Ca^2+^. Further examination into this phenomenon revealed that, in addition to Ca^2+^, posttranslational modifications regulate the enzyme activity of eNOS^3,4^; these modifications include phosphorylation and O-GlcNAcylation^3^. Similar to protein phosphorylation, cytokines and glucose metabolism can regulate protein function via O-GlcNAcylation^5^. In response to various types of stressors, cells quickly increase glucose uptake, and the ability of cells to transport glucose is related to the ability of cells to react to stressors and ultimately survive under deleterious conditions^6–8^. It has been clarified that the O-GlcNAc levels of nuclear and cytoplasmic proteins in multiple cell lines increase rapidly and dynamically under cellular stress^9^.

The literature^10,11^ indicates that hyperosmosis can affect cardiovascular function, but the underlying mechanisms remain unclear. It was reported^12^ that cardiovascular disease occurs as a result of hyperosmotic stress-induced endothelial tyrosine phosphorylation that deteriorates barrier performance. eNOS was identified as a key molecule in the process of endothelial barrier protection^13^. The effect of hypertonic stress on vascular endothelial eNOS activity and the mechanism involved have not yet been reported. In this article, we mainly studied the effect of hypertonic stress on eNOS O-GlcNAc levels and discussed the possible mechanism.

## Materials and methods

### Materials

DMEM (Invitrogen, Carlsbad, CA, USA), fetal bovine serum (Gibco, USA), and anti-eNOS (Cat No. 32027, Cell Signaling Technology, USA), anti-AMPK (Cat No. 9158, Cell Signaling Technology, USA), anti-O-GlcNAc (Cat No. 05-1245, Sigma, USA), anti-O-linked N-acetylglucosamine (GlcNAc) transferase (OGT) (Cat No. ab177941, Abcam, England) anti-protein O-GlcNAcase (OGA) (Cat No. ab124807, Abcam, England), goat anti-mouse IgG (Cat No. SA00001-1, Proteintech, USA), and goat anti-rabbit IgG (Cat No. SA00001-2, Proteintech, USA) antibodies were used. Other reagents are mentioned as they appear in the article.

### Plasmid construction

The wild-type (WT) human eNOS cDNA sequence (provided by Dr. Yong Xia) was subcloned into pCDN3.1 (His tag). eNOS-T866A is a point mutant of eNOS-WT constructed by site-directed mutagenesis (Thr866 mutated to Ala). The mutation was performed by TransGen (TransGen Biotech, Beijing, China) and confirmed by direct sequencing.

### Measurement of NO

The NO content of the culture medium was measured with a modified Griess reaction method (Beyotime) following the product instructions.

### Culture of BAECs and HEK293 cells

Bovine aortic endothelial cells (BAECs) and the human embryonic kidney cell line HEK293 (kindly provided by Dr. Yong Xia) were cultured with DMEM supplemented with 10% FBS and incubated in an incubator with 5% CO_2_ and 95% O_2_. BAECs from passages 6 to 10 and with 80% to 90% purity were used for experiments. Cells were randomly assigned various treatments, including hypertonic stress (130 mM, 150 mM, 170 mM, and 190 mM NaCl for 24 h), heat stress (40 °C for 2 h), oxidative stress (200 mM hydrogen peroxide for 24 h or 200 mM ethanol for 24 h), hypoxia (1% O_2_ for 12 h) and acidosis (pH 7, pH 6.5 for 6 h). The control group was cultured with conventional medium for the same time.

### Transfection

AMPKα1-specific siRNA sequences (5′-GAUCCAUCAUAUAGCUCAAdTdT-3′ and 3′-UUGAGCUAUAUGAUGGAUCdTdT-5′) were constructed; the siRNA serial number is the one reported in the article. Cells were transfected according to the manufacturer's protocol. Briefly, siRNA (final concentration 100 nM in Opti-MEM) was delivered into the cells with Lipofectamine 3000 (Invitrogen) reagent. At 48 h after transfection, the cells were processed for subsequent experiments. For cell transfection, BAECs cultured in a 100-mm dish were transfected with plasmid encoding eNOS-WT and eNOS-T866A using Lipofectamine 3000 (Invitrogen) following the manufacturer’s instructions of use and analyzed 48 h after transfection. HEK293 cells in 6-well cell culture plates were transfected with plasmid encoding eNOS-WT or eNOS mutants using Lipofectamine 2000 (Invitrogen) following the manufacturer’s instructions of use and analyzed 24 h after transfection^23^.

### Protein expression and purification

In HEK293 cells and BAECs, protein purification was carried out in accordance with the procedures of the His Tag Protein Kit (Beyotime Biotechnology, Jiangsu, China). One milliliter of a well-mixed storage solution containing 50% His tag protein purification resin was centrifuged, and the supernatant was aspirated. The pellet was resuspended and mixed with 0.5 mL of lysate (without inhibitor) before 60 µL of cell stock was added to the pretreated resin in accordance with the His-tagged protein purification resin (12–20 mg/mL purified protein ratio) and washed with lysis buffer to a volume 300 µL. The EP tube was slowly rotated at 4 °C and mixed for 2 h.

### Immunoblotting

The supernatant was obtained when the cells were mixed with lysis buffer at 4 °C for 1.5 h and then centrifuged (12,000×*g*, 10 min). The protein concentration was determined by Coomassie blue staining. An appropriate amount of loading buffer was added according to the volume, and the samples were boiled to denature proteins before and an appropriate amount was used for western blotting. Proteins were separated by SDS-PAGE, transferred to PVDF membranes (Bio-Rad, USA) and blocked with 5% skim milk (Oxoid, England) for 1.5 h at room temperature. The membrane was incubated with the primary antibody overnight at 4 °C, washed, incubated with secondary antibody for 1.5 h at room temperature, and washed again, and the immunoreactive protein bands were detected using a chemiluminescence detector (Beyotime, China).

### Statistical evaluation

All data are presented as the means ± SEM. The differences in all the data were analyzed by two-way ANOVA, and then unpaired t-tests were performed by GraphPad Prism version 8.0. P < 0.05 was considered statistically significant.

## Results

### Physical and chemical factors change the modification level of eNOS O-GlcNAc

**Figure 1:**
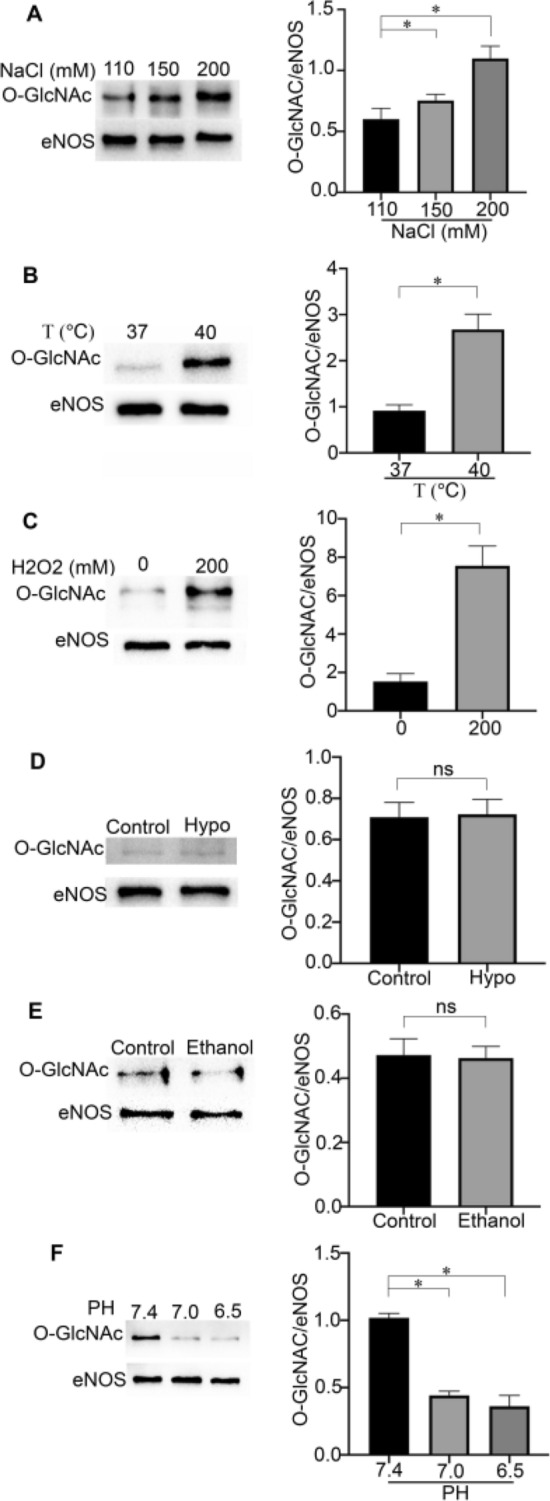
Effects of physical and chemical factors on eNOS O-GlcNAc in HEK293 cells transfected with WT-eNOS. (**A**) High NaCl increased the ratio of O-GlcNAc/eNOS in HEK293S cells. Typical images and statistical graphs are shown, n = 3. (**B**) Hyperthermia increased the ratio of O-GlcNAc/eNOS in HEK293 cells. Typical images and statistical graphs of the ratio of O-GlcNAc/eNOS in HEK293 cells, n = 3. (**C**) Hydrogen peroxide increased the ratio of O-GlcNAc/eNOS in HEK293 cells. Typical images and statistical graphs are shown, n = 3. (**D**) Hypoxia had no effect on the ratio of O-GlcNAc/eNOS in HEK293 cells. Typical images and statistical graphs are shown, n = 3. (**E**) Ethanol had no effect on the ratio of O-GlcNAc/eNOS in HEK293S cells. Typical images and statistical graphs are shown, n = 3. (**F**) The decrease in pH reduced the ratio of O-GlcNAc/eNOS in HEK293 cells. Typical western blot images are shown in the left panel. The statistical results are shown in the right panel; Full-length blots are presented in Supplementary Figure S1A, S2B, S3C, S4D, S5E, S6F. n = 3. *P < 0.05. ns indicates no significance.

To confirm that physical and chemical factors affect eNOS O-GlcNAcylation, western blotting was performed to detect O-GlcNAcylation under various physical and chemical treatments. The results showed that compared with the normal NaCl group, the 200 mM NaCl group showed increased eNOS O-GlcNAc content (Fig. 1A). Similar to hypertonic stress, compared with the normal temperature of 37 °C for cell growth, heat stress increased the eNOS O-GlcNAc content (Fig. 1B), as did 200 mM hydrogen peroxide (Fig. 1C), which indicated that hypertonic stress, hyperthermia and hydrogen peroxide may have similar effects on eNOS activity. However, the ratio of eNOS O-GlcNAc to eNOS did not change in cell subjected to hypoxia or ethanol treatment (Fig. 1D,E), which indicated that neither hypoxia nor ethanol affected eNOS O-GlcNAc; this translates to not all physical and chemical factors exerting similar effects as those induced by hypertonic stress. Interestingly, under a lower pH, the eNOS O-GlcNAc/eNOS ratio was significantly reduced (Fig. 1F). These results strongly indicated that different physical and chemical factors have different effects on eNOS O-GlcNAc.

### Hypertonic stress results in an increase in eNOS O-GlcNAc levels via Thr866

**Figure 2:**
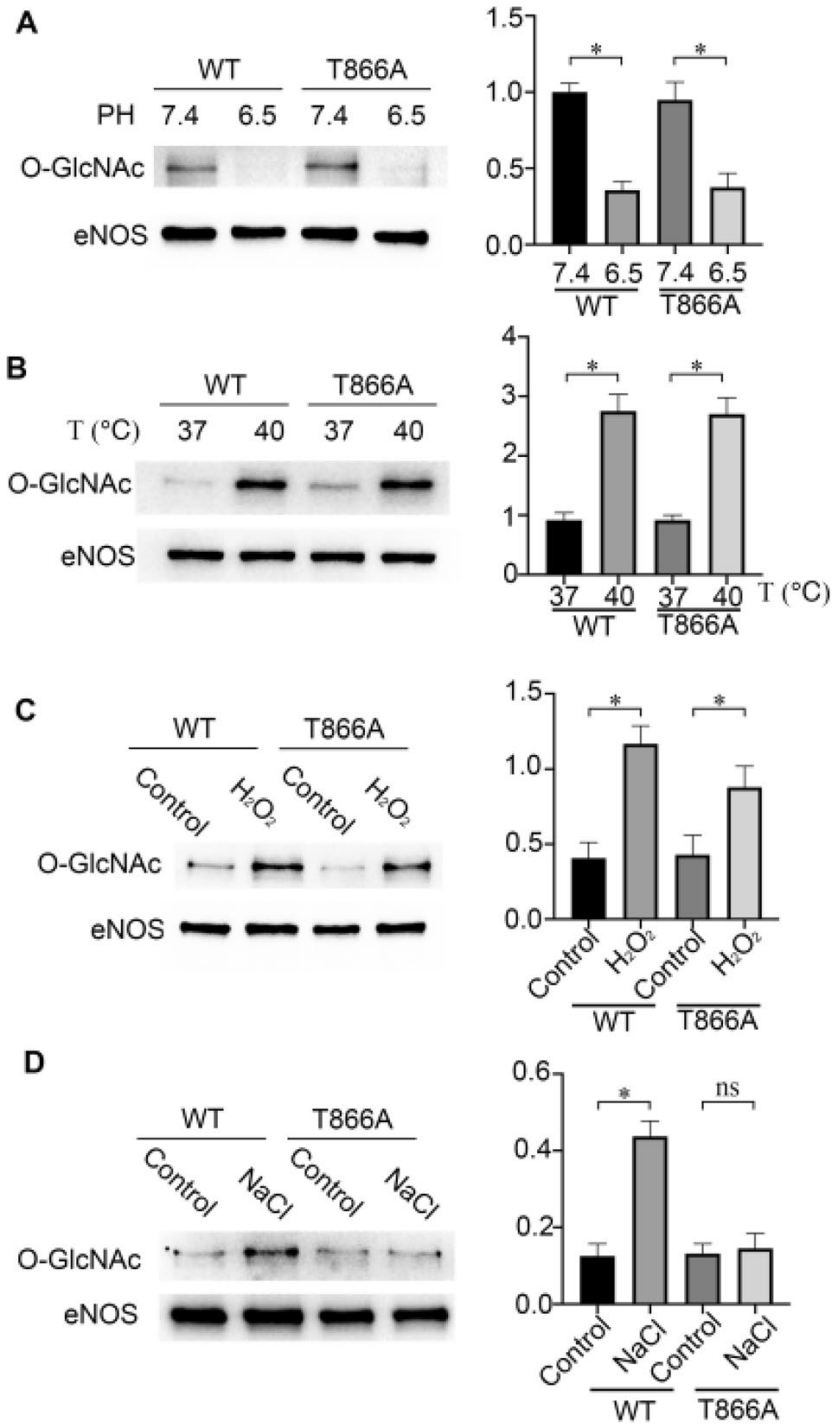
Changes in the levels of O-GlcNAc/eNOS in HEK293 cells (**A**) After decreasing PH, changes in the expression of O-GlcNAc/eNOS in HEK293 cells. Typical images and statistical graphs are shown, n = 3. (**B**) Changes in the levels of O-GlcNAc/eNOS in HEK293 cells under hyperthermic conditions. Typical images and statistical graphs are shown, n = 3. (**C**) Changes in the levels of O-GlcNAc/eNOS in HEK293 cells treated with hydrogen peroxide. Typical images and statistical graphs are shown, n = 3. (**D**) Changes in the levels of O-GlcNAc/eNOS in HEK293 cells treated with NaCl (200 mM). Typical images and statistical graphs are shown, Full-length blots are presented in Supplementary Figure S7A, S8B, S9C, S10D. n = 3. *P < 0.05; ns indicates no significance.

Previous research by our group showed that Thr866 is the key site for low-glucose mediated regulation of eNOS O-GlcNAc. To explore whether other physical and chemical factors act through Thr866 in eNOS, the effects of the abovementioned physical and chemical factors on the levels of eNOS O-GlcNAc after Thr866 was mutated were detected by western blot. The results showed that eNOS O-GlcNAc levels still changed in response to acidosis, hyperthermia and hydrogen peroxide-induced oxidative stress in cells with the Thr866 site mutation (Fig. 2A–C). However, O-GlcNAc levels did not change under hypertonic stress compared with those of the control (Fig. 2D). These results indicated that eNOS Thr866 might be an O-GlcNAcylation site activated by hypertonic stress.

### Hypertonic stress causes upregulation of OGT expression and activation of AMPK

**Figure 3:**
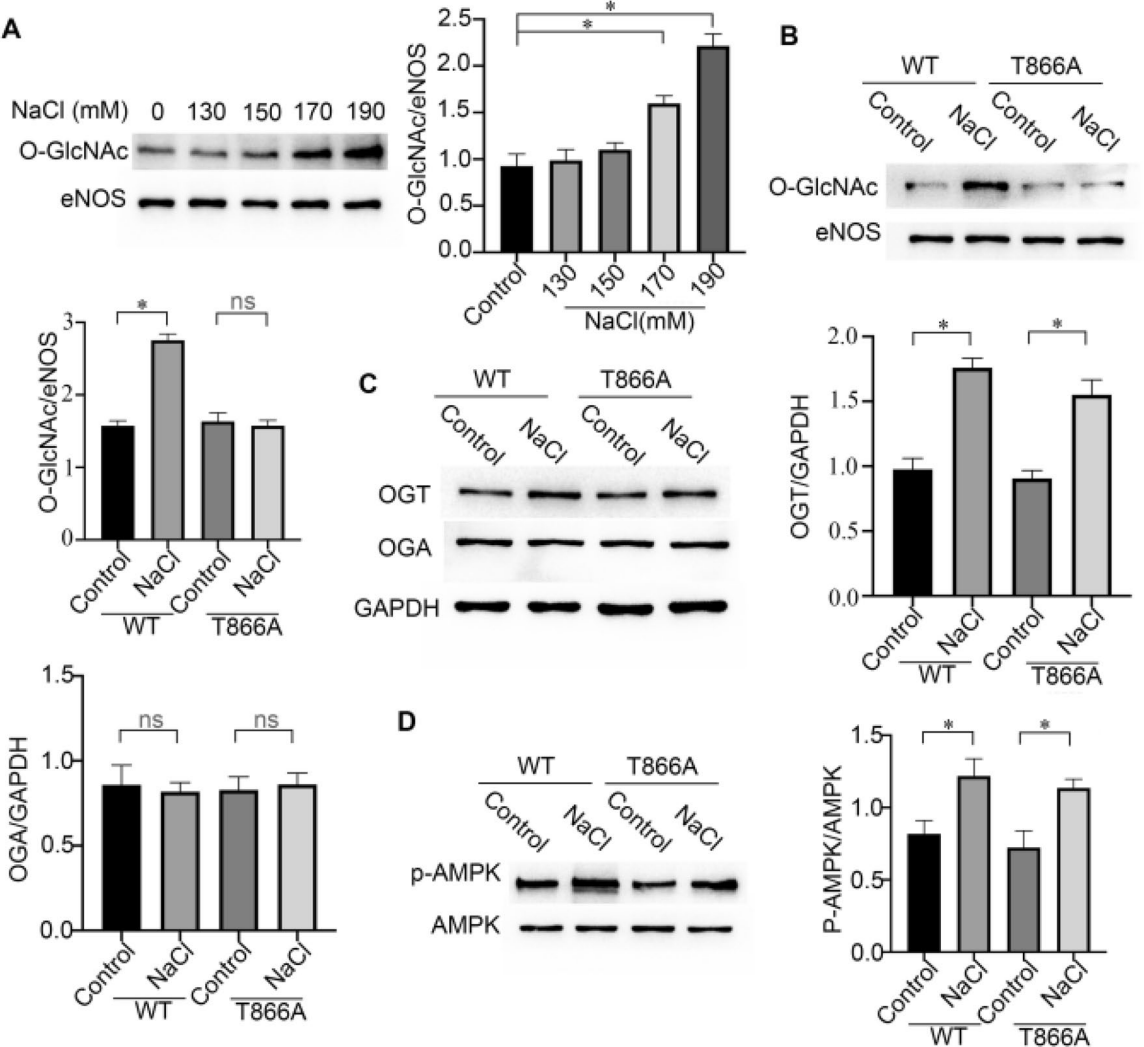
Hypertonic stress causes upregulation of OGT expression and activation of AMPK in cells transfected with His-tagged WT-eNOS or T866A-eNOS. (**A**) Changes in the levels of O-GlcNAc/eNOS in BAECs treated with 110 to 190 mM NaCl. Typical images and statistical graphs are shown, n = 3. (**B**) Changes in the levels of O-GlcNAc/eNOS in BAECs treated with NaCl (190 mM). Typical images and statistical graphs are shown, n = 3. (**C**) Changes in the expression of OGT and OGA in BAECs treated with NaCl (190 mM). Typical images and statistical graphs are shown, n = 3. (**D**) Changes in the ratio of p-AMPK/AMPK in BAECs treated with NaCl (190 mM). Typical images and statistical graphs are shown, Full-length blots are presented in Supplementary Figure S11A, S12B, S13C, S14D. n = 3. *P < 0.05, ns indicates no significance.

This phenomenon was observed in HEK293 cells. To determine whether there were changes in O-GlcNAc/eNOS levels in endothelial cells, the hypertonic treatment was repeated with BAECs. The results of primary endothelial cells were consistent with those of HEK293 cells. When BAECs were exposed to 190 mM NaCl, the levels of O-GlcNAc/eNOS increased compared with those in cells cultured in normal NaCl (Fig. 3A). The level of O-GlcNAc on eNOS showed no changes in BAECs transfected with T866A-eNOS (Fig. 3B). The O-GlcNAcylation cycle is regulated by two enzymes, OGT and OGA^14^. To further explore the regulatory mechanism of eNOS O-GlcNAc under hypertonic conditions, proteins in cells subjected to hypertonic treatment were evaluated by western blotting. The results suggested that hypertonic stress increased the OGT content, while the OGA content remained unchanged (Fig. 3C). AMPK, a cellular sensor, could participate in the expression of O-GlcNAc protein. Reportedly^15^. To confirm the relationship between AMPK and O-GlcNAc, the levels of p-AMPK and AMPK were detected using western blotting. After cells were incubated for 24 h in hypertonic conditions, the p-AMPK/AMPK ratio was increased (Fig. 3D). These results suggested that hypertonic stress can upregulation OGT expression and activate AMPK.

### Hypertonic stress increases eNOS O-GlcNAc levels via upregulation of OGT expression and activation of AMPK

**Figure 4:**
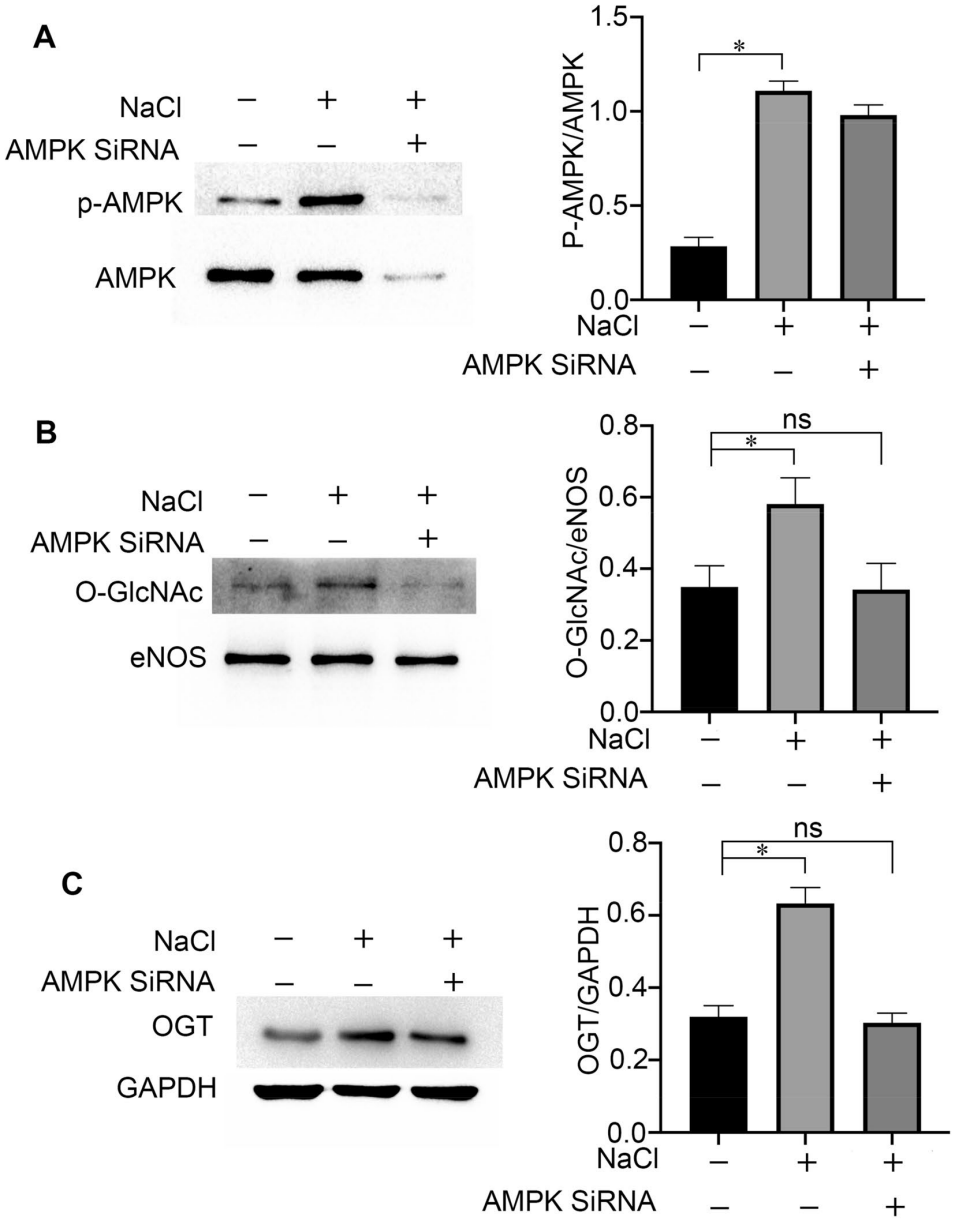
Hypertonic stress increased the levels of eNOS O-GlcNAc and OGT in an AMPK-dependent manner. (**A**) Changes in the ratio of p-AMPK/AMPK in BAECs treated with NaCl (190 mM). Typical images and statistical graphs are shown, n = 3. (**B**) Changes in the levels of O-GlcNAc/eNOS in BAECs treated with NaCl (190 mM). Typical images and statistical graphs are shown, n = 3. (**C**) Changes in the ratio of OGT/GAPDH in BAECs treated with NaCl (190 mM). Typical images and statistical graphs are shown, Full-length blots are presented in Supplementary Figure S15A, S16B, S17C. n = 3. *P < 0.05, ns indicates no significance.

To confirm that the increased eNOS O-GlcNAc levels induced by hypertonic stress is the result of activation of AMPK and upregulation of OGT expression, the cells were first subjected to AMPKα1 knockdown, and then the levels of O-GlcNAc, OGT, eNOS, p-AMPK and AMPK were detected by western blot. After cells were incubated for 24 h with hypertonic stress, the ratio of O-GlcNAc/eNOS, OGT/GAPDH and p-AMPK/AMPK were increased. However, as the figures show, the levels of O-GlcNAc, OGT, and p-AMPK were reversed upon AMPKα1 knockdown (Fig. 4 A, B, C). These results strongly indicated that hypertonic stress increased the levels of O-GlcNAc/eNOS and OGT in an AMPK-dependent manner.

### An inactivating mutation at eNOS Thr866 prevents activation of O-GlcNAcylation by OGT under hypertonic stress

**Figure 5:**
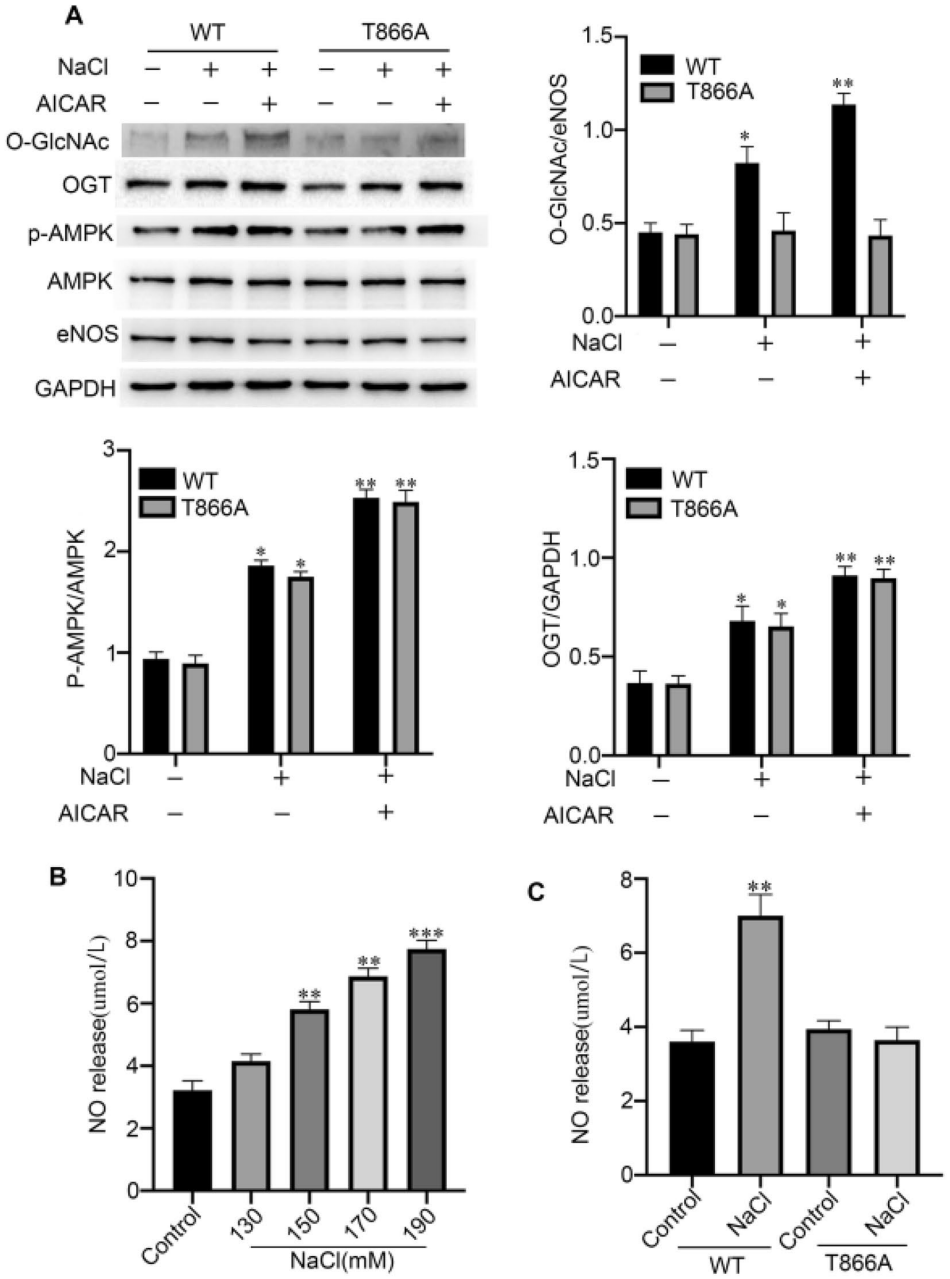
An inactivating mutation at eNOS Thr866 prevents activation of O-GlcNAcylation by OGT under hypertonic stress. (**A**) Changes in the level of O-GlcNAc/eNOS and the ratios of p-AMPK/AMPK and OGT/GAPDH in BAECs treated with NaCl (190 mM) and/or AICAR (activator of AMPK). Typical images and statistical graphs are shown, n = 3. (**B**) NO release from BAECs cultured under hypertonic conditions (110 to 190 mM NaCl). (**C**) NO release from BAECs transfected with WT-eNOS or T866A-eNOS and cultured in the hypertonic medium (190 mM NaCl). Full-length blots are presented in Supplementary Figure S18A. *P < 0.05; **P < 0.01; ***P < 0.001.

To further confirm that hypertonic stress leads to an increase in eNOS O-GlcNAc levels via modification at the Thr866 site, AICAR(activator of AMPK)-treated cells were processed for western blot to detect the levels of O-GlcNAc, OGT, eNOS, p-AMPK and AMPK. O-GlcNAc and OGT levels were increased in AICAR-treated cells; however, this increase of O-GlcNAc was reversed in cells transfected with T866A-eNOS (Fig. 5A). In addition, to demonstrate the effect of increased O-GlcNAc levels by hypertonic stress on the content of NO produced by eNOS, we measured the NO content in the cell culture medium under high NaCl. The levels of NO were increased in a concentration-dependent manner (Fig. 5B), and the levels of NO in the T866A-eNOS group were lower than those in the WT group (Fig. 5C). These results strongly indicated that hypertonic stress induces increased NO synthesis by eNOS through posttranslational addition of )-GlcNAc at Thr866.

## Discussion

Environmental, physiological and chemical stresses all cause signal transduction events, which ultimately lead to changes in the levels of compounds present, thus weakening the effects of harmful stressors on cells and counteracting signals that promote apoptosis or necrosis^16,17^. Most of the mechanisms involved in these signal transduction pathways are related to protein phosphorylation^18,19^. In this study, we showed that the O-GlcNAc modification in mammalian vascular endothelial cells increased rapidly and dynamically after exposure to noxious physical, chemical and biological stimuli, which indicated that O-GlcNAcylation is a key molecule in the cellular respond to stress^9^.

This study shows that O-GlcNAcylation can respond to a variety of physical and chemical factors, but its regulatory mechanism is quite different. For example, hypertonic stress, heat stress, and other stressors increased the level of the eNOS with the O-GlcNAc modification, whereas acidosis leads to reductions in the eNOS O-GlcNAc modification. Ser1177 participates in mediating the activation of eNOS under hypertonic stress^20^, though the role of O-GlcNAcylation regulation in hypertonic regulation is still poorly understood. A large number of studies have shown that O-GlcNAcylation plays a novel role in the cardiovascular system^21,22^. In previous work, it was found that Thr866 is the key site for low-glucose-mediated regulation of eNOS O-GlcNAc levels^23^. In this study, we explored the effects of hypertonic stress on the levels of eNOS O-GlcNAc and observed that the Thr866 mutation prevented the eNOS/O-GlcNAc modification. These results indicated that Thr866 is also the key site for hypertonic stress regulation of eNOS O-GlcNAc.

Both OGT and AMPK are regulated by nutrient-sensitive pathways and synergistically affect dynamic metabolic balance and intracellular life processes. The regulation of OGT and AMPK could prevent cells from experiencing injury due to metabolic stressor such as glucose deprivation, endoplasmic reticulum stress and H_2_O_2_-induced mitochondrial stress^24^. Similar to phosphorylation, O-GlcNAcylation might also be catalyzed by OGT through kinases such as AMPK and then regulate proteins (including nuclear, cytoplasmic, and mitochondrial proteins) via Ser-/Thr-specific posttranslational modifications^25^. OGT and AMPK target a variety of intracellular proteins, and in addition to regulating nutrient-sensitive intracellular processes, their net role is to protect cells from metabolic stress^15^. Studies have reported that OGT and AMPK could be directly intermodulated, and phosphorylation of OGT was related to the activity of AMPK^15^. Increased AMPK expression is a prerequisite for OGT activity during glucose deprivation^26^. Our results show that hypertonicity causes acute activation of AMPK, thereby increasing the expression level of OGT and, consequently, the levels of eNOS O-GlcNAc, but these changes are reversed after AMPKα1 knockdown. These results strongly indicated that hypertonic stress increased the levels of eNOS O-GlcNAc and OGT in an AMPK-dependent manner.

To eliminate the interference of protein components other than eNOS, we used an affinity precipitation method to purify eNOS from total cell protein. The current data demonstrate that hypertonic stress promoted the O-GlcNAc modification at Thr866 of eNOS, thus enhancing eNOS activity. This regulation of eNOS O-GlcNAc levels may be caused by AMPK activation to the observed increase in the level of OGT expression. In vitro experiments showed that hypertonic stress induces an increase in the levels of eNOS O-GlcNAc, thereby protecting vascular endothelial cells from external stressors.

This study has limitations. Phosphorylation is another important posttranslational modification of eNOS, but we did not explore whether phosphorylation contributes to its activity. In addition, we have not ruled out the role of Ser1177, a positive regulatory site for eNOS that has been reported in previous studies^20^.

## Conclusion

In summary, the present study shows that the Thr866 site plays an important role in O-GlcNAcylation induced by hypertonic stress.Hypertonic stress can upregulate the expression of OGT, which is dependent on AMPK. When AMPK was knocked out, the upregulation of OGT expression and increased O-GlcNAc modifications induced by hypertonic stress were reversed. Therefore, our study broadens the persperctive regarding the analysis of glycosylation of eNOS and may offer a new field of vision for the effect of hypertonic stress on endothelial cells.

## Supplementary Information

Supplementary Information.
