# Supplementary material for: Hypertonic stress modulates eNOS function through O-GlcNAc modification at Thr-866
Source: Sci Rep. 2021 May 28;11:11272. doi: 10.1038/s41598-021-90321-4 (PMC8163736; doi:10.1038/s41598-021-90321-4)
Supplement: Supplementary file 1 — Supplementary Information. [file 41598_2021_90321_MOESM1_ESM.pdf]

Supplementary Information for

Hypertonic stress modulates eNOS function through O-GlcNAc modification at Thr-866

Chang Li [a, b], An He [a], Yongzheng Guo [a, b], Xiyang Yang [a, b], Minghao Luo [a, b], Zhe Cheng [a, b], Xianglong Huang [a, b], Yong Xia [a, b, c] *, Suxin Luo [a] *

[a] Division of Cardiology, The First Affiliated Hospital of Chongqing Medical University, Chongqing 400016, China

[b] Institute of Life Science, Chongqing Medical University, Chongqing 400016, China

[c] Davis Heart and Lung Research Institute, Division of Cardiovascular Medicine, Department of Molecular and Cellular Biochemistry, The Ohio State University College of Medicine, 473 West 12th Avenue, Columbus, OH, 43210, USA

*Corresponding authors.

E-mail addresses: yongxia_68@163.com (Yong Xia), suxinluo1971@163.com (Suxin Luo)

Supplementary Figure Legends

Supplementary Figure S1. Full western blot pictures shown in Figure 1A.

Supplementary Figure S2. Full blots for blots shown in Figure 1B.

Supplementary Figure S3. Full blots for blots shown in Figure 1C.

Supplementary Figure S4. Full blots for blots shown in Figure 1D.

Supplementary Figure S5. Full blots for blots shown in Figure 1E.

Supplementary Figure S6. Full blots for blots shown in Figure 1F.

Supplementary Figure S7. Full blots for blots shown in Figure 2A.

Supplementary Figure S8. Full blots for blots shown in Figure 2B.

Supplementary Figure S9. Full blots for blots shown in Figure 2C.

Supplementary Figure S10. Full blots for blots shown in Figure 2D.

Supplementary Figure S11. Full blots for blots shown in Figure 3A.

Supplementary Figure S12. Full blots for blots shown in Figure 3B.

Supplementary Figure S13. Full blots for blots shown in Figure 3C.

Supplementary Figure S14. Full blots for blots shown in Figure 3D.

Supplementary Figure S15. Full blots for blots shown in Figure 4A.

Supplementary Figure S16. Full blots for blots shown in Figure 4B.

Supplementary Figure S17. Full blots for blots shown in Figure 4C.

Supplementary Figure S18. Full blots for blots shown in Figure 5A.

The red boxes represent the blots used in the Figures

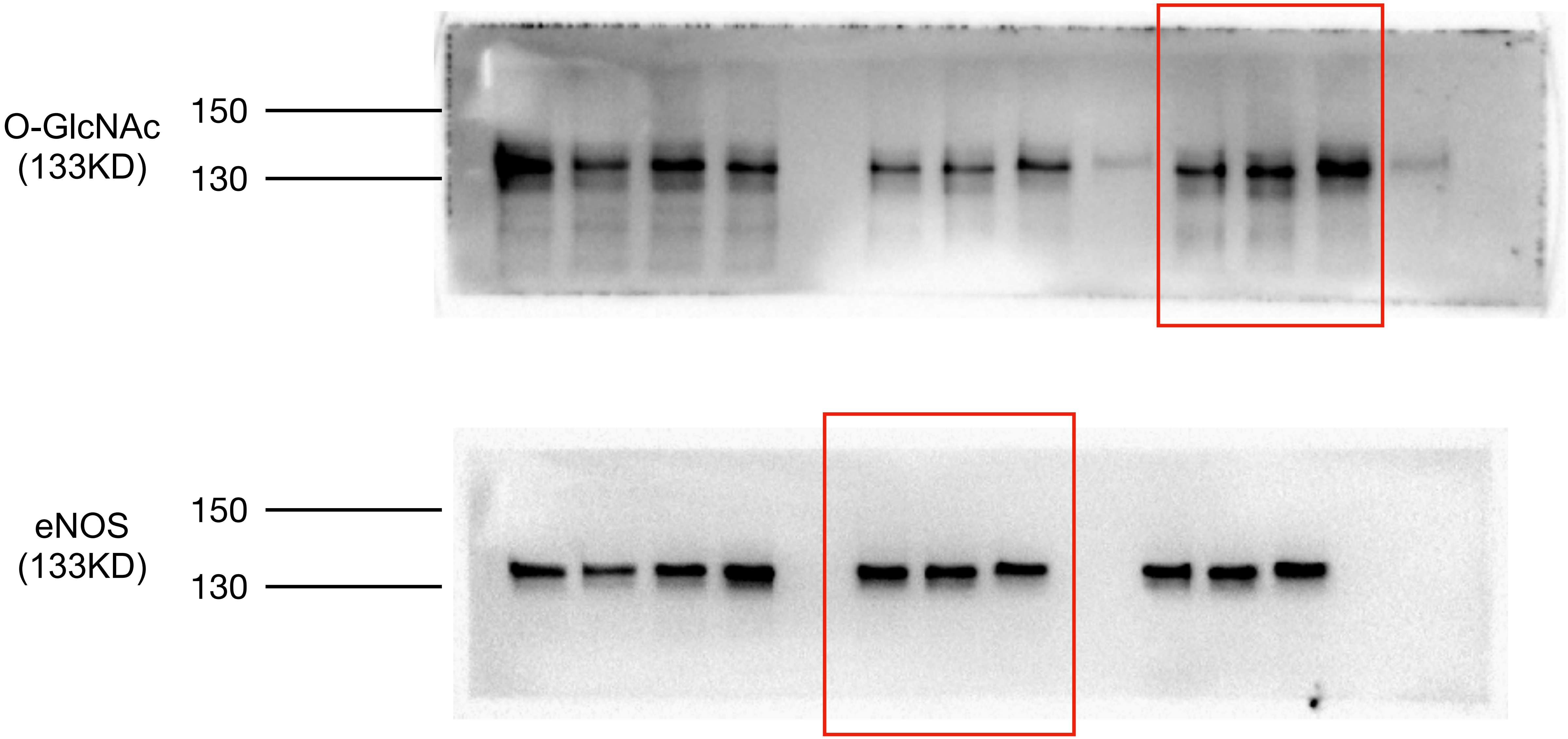

Supplementary Figure S1

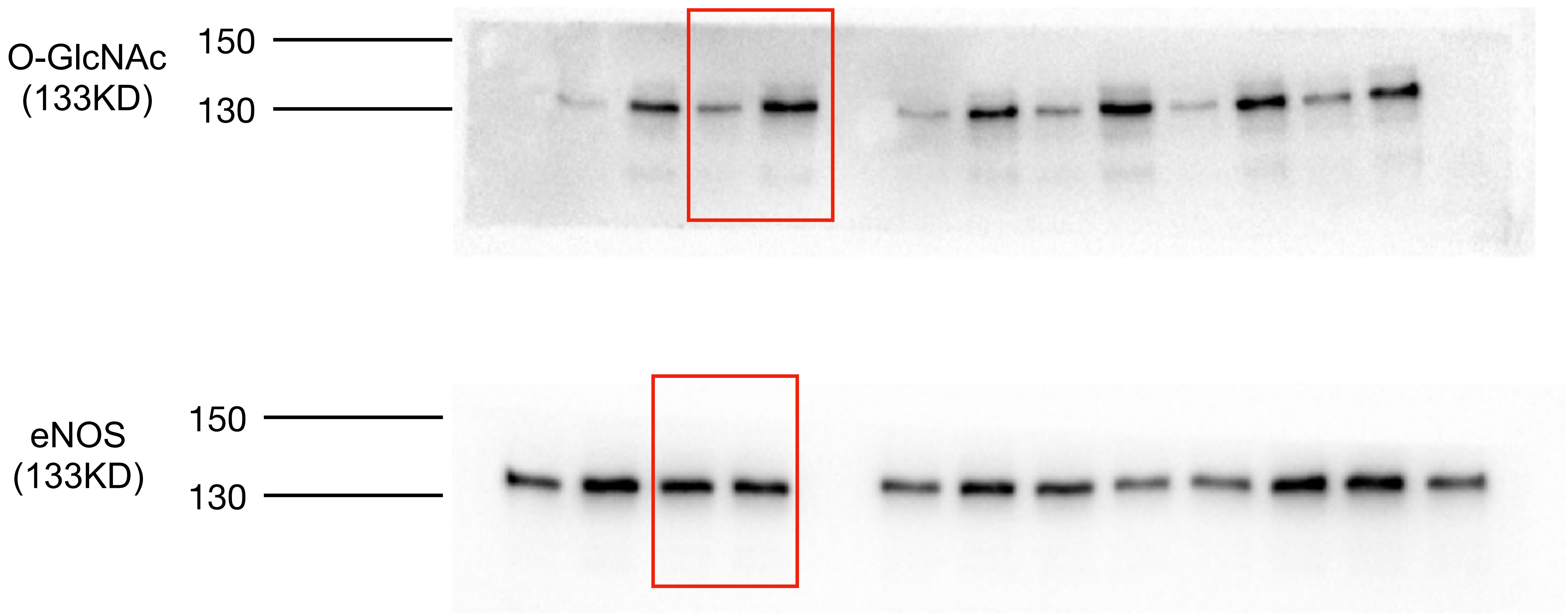

Supplementary Figure S2

The reasons for why the original blots provided are tightly cropped are following: there only exist one target proteins which is purified eNOS O-GlcNAc (up)/eNOS (down) needs to be determined by immunoblotting. And, the molecular weight of eNOS is 133KD. Hence we only reserve part of PVDF membrane (molecular weight range: 130-150 Kda) for suiting our target proteins. In summary, this is the reason why this original blot is tightly cropped.

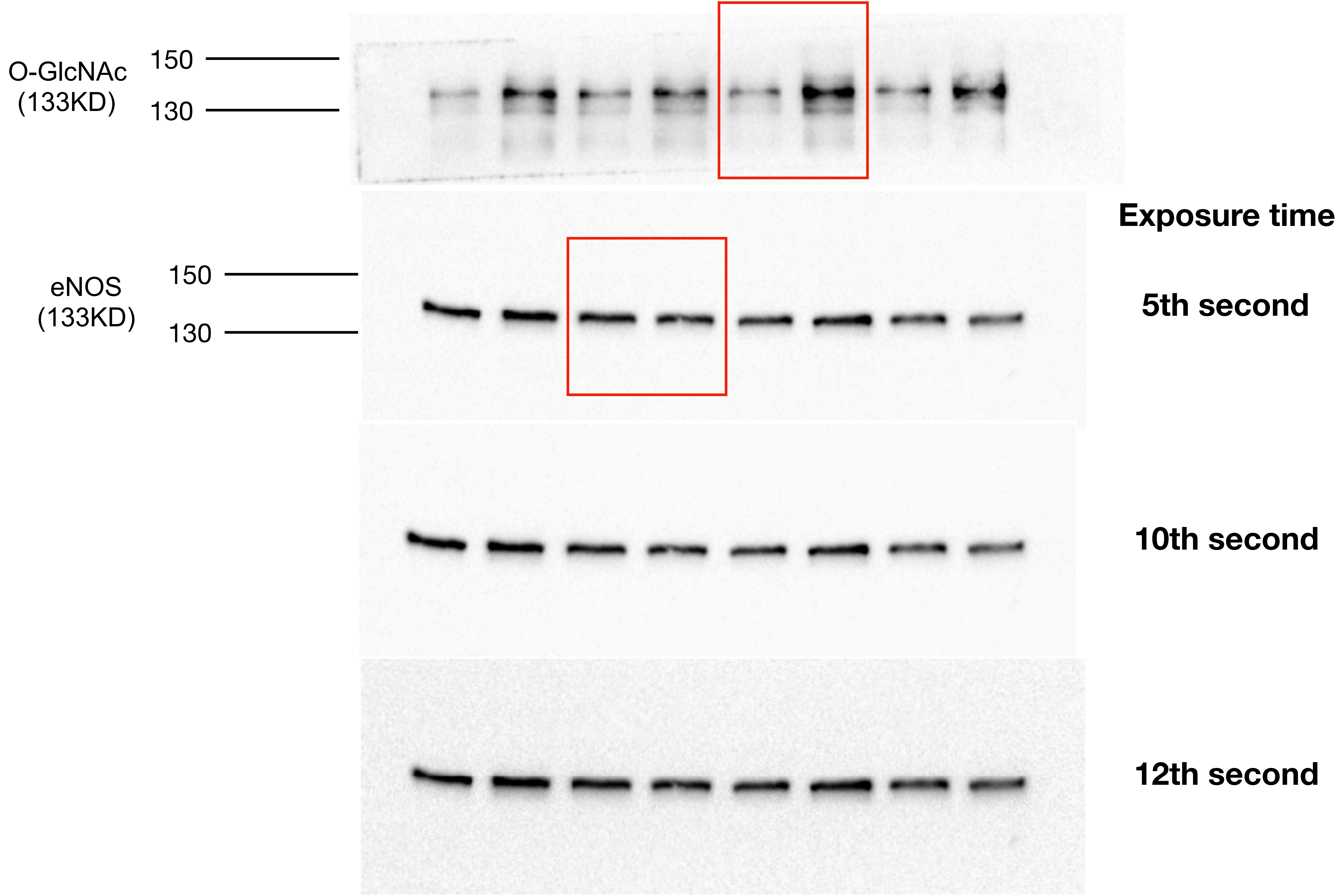

Supplementary Figure S3

Two proteins in a group, repeat the sample four times on a piece of membrane. Although the swimming lanes are different, I chose the more typical group.We provide exposure images at multiple time points.

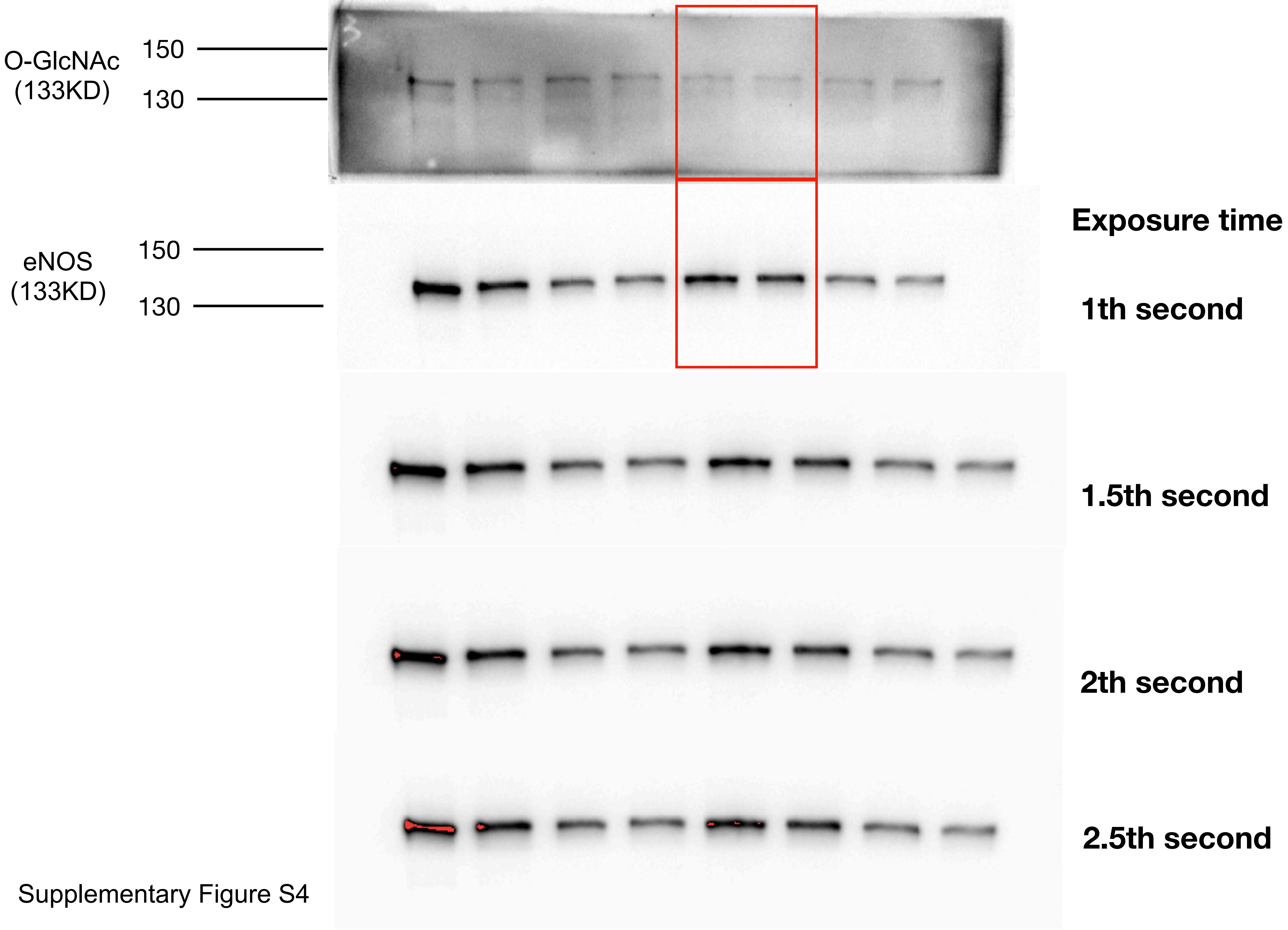

Supplementary Figure S4

We provide exposure images at multiple time points.

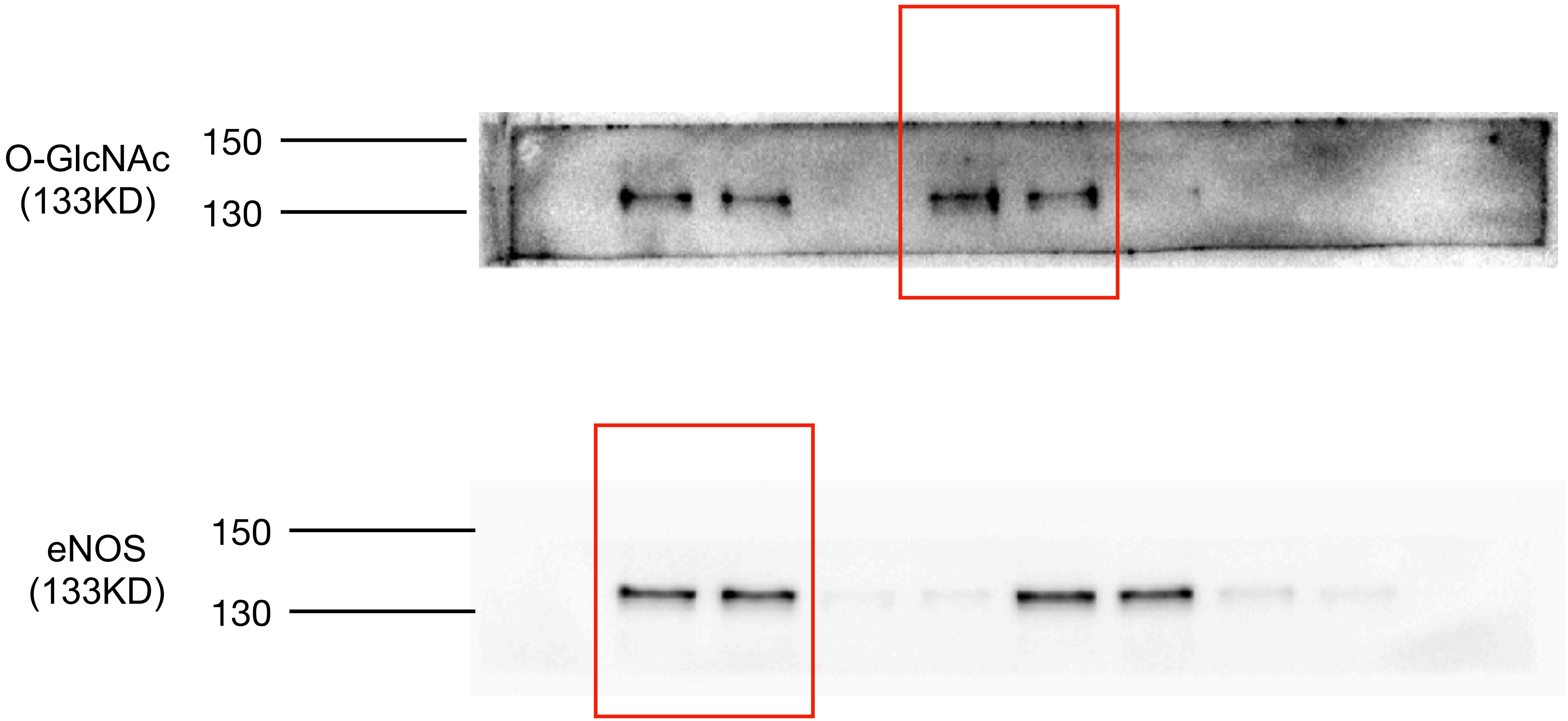

Supplementary Figure S5

Two proteins in a group, repeat the sample two times on a piece of membrane.
Although the swimming lanes are different, I chose the more typical group.

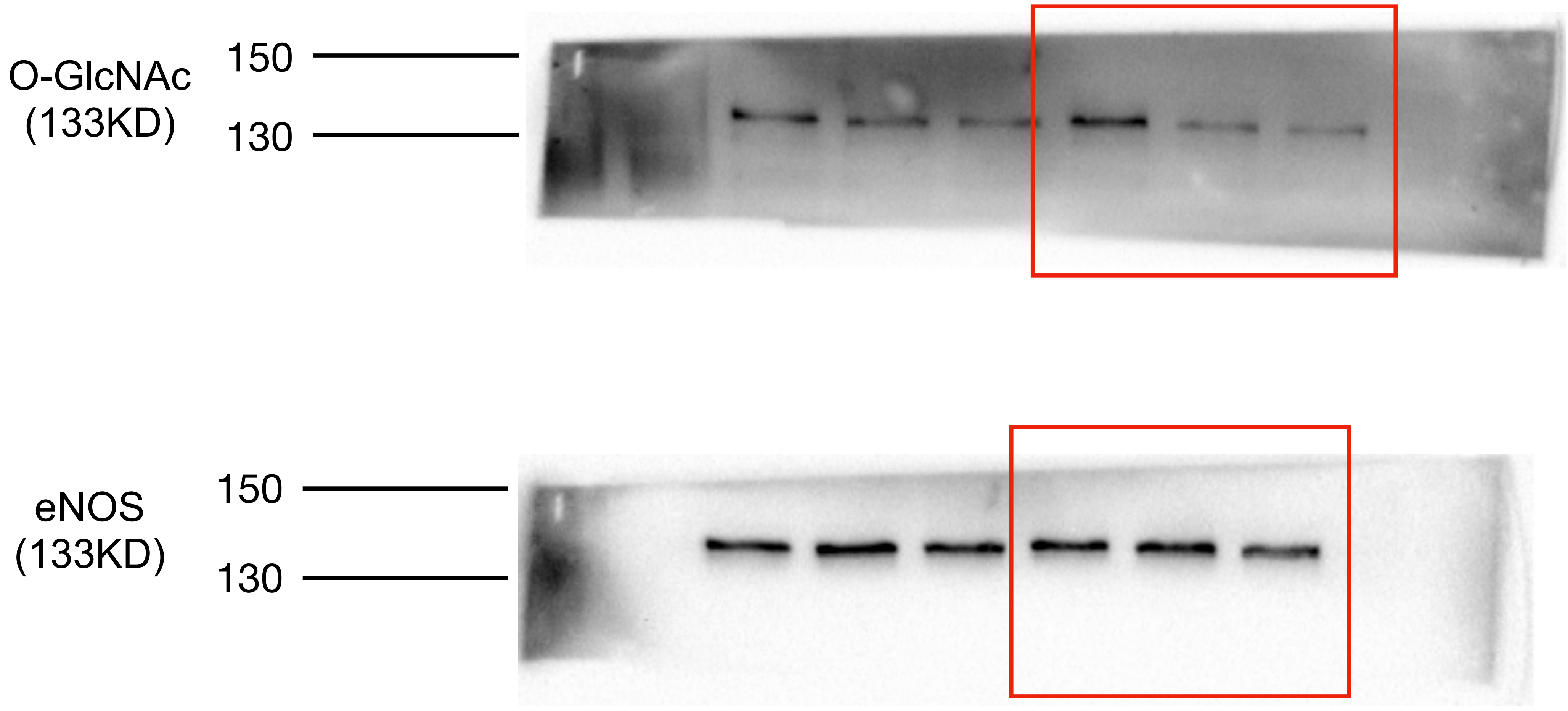

Supplementary Figure S6

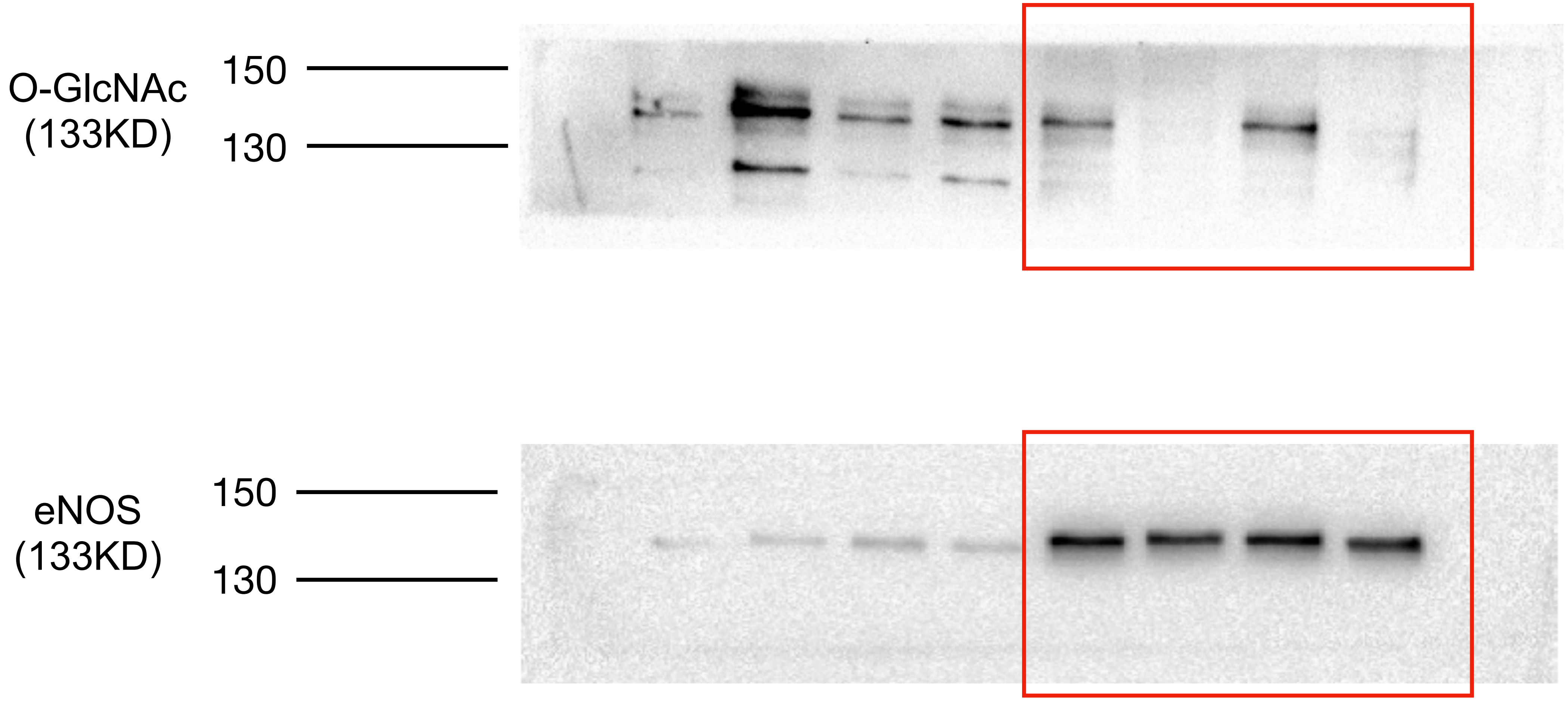

Supplementary Figure S7

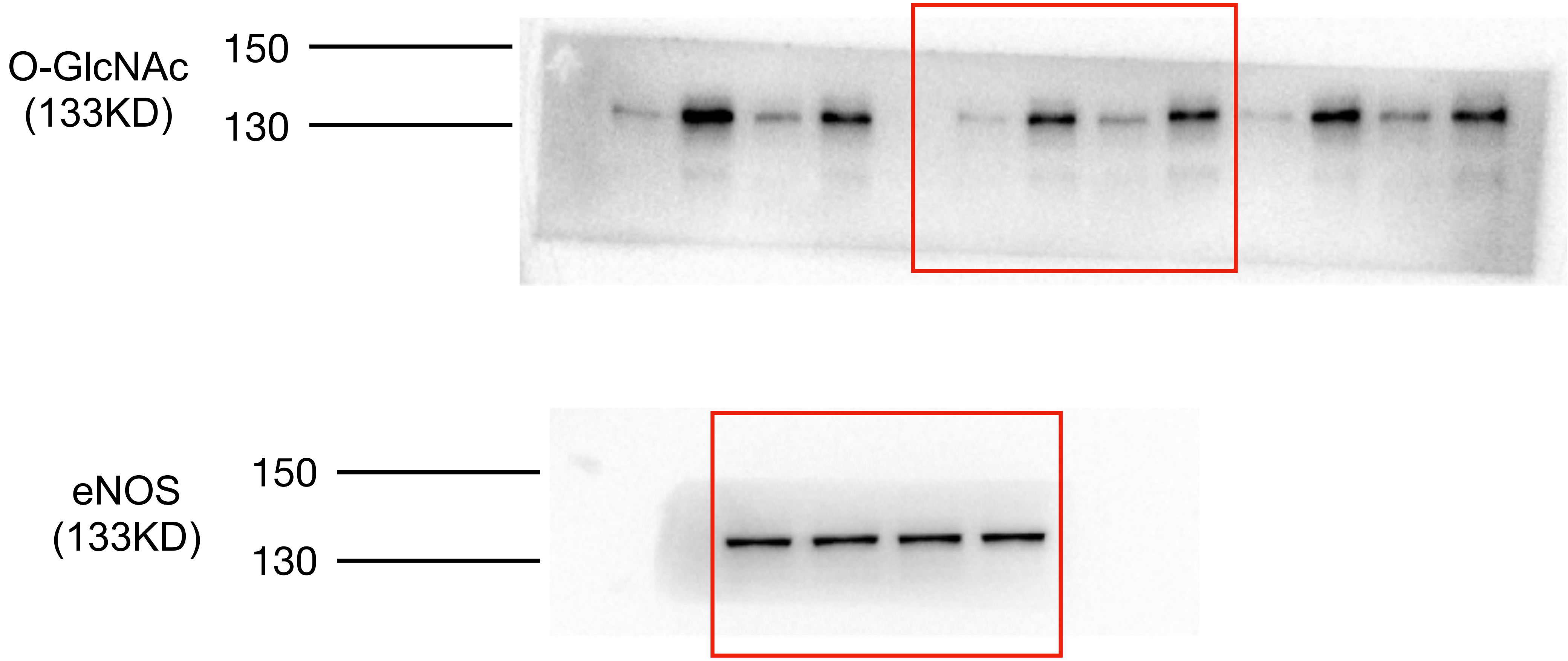

Supplementary Figure S8

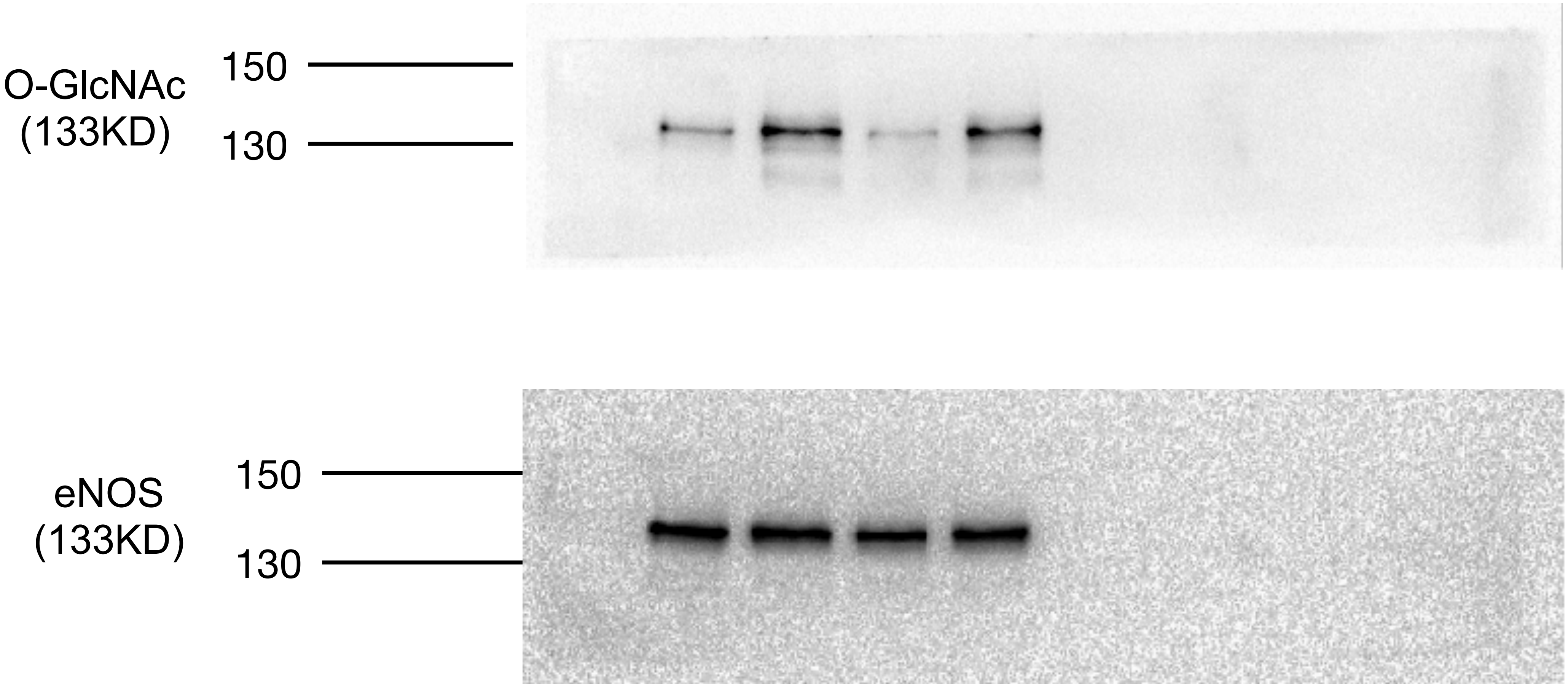

Supplementary Figure S9

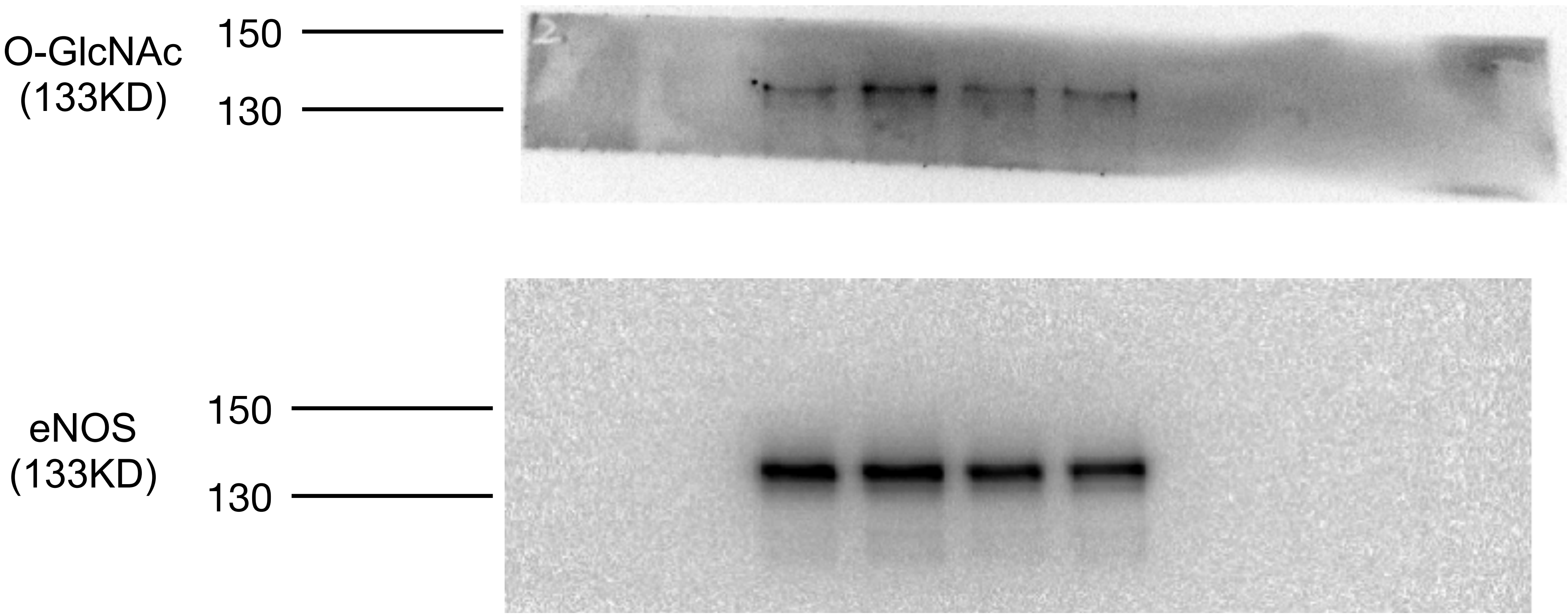

自动曝光 无其他时间点图片

Supplementary Figure S10

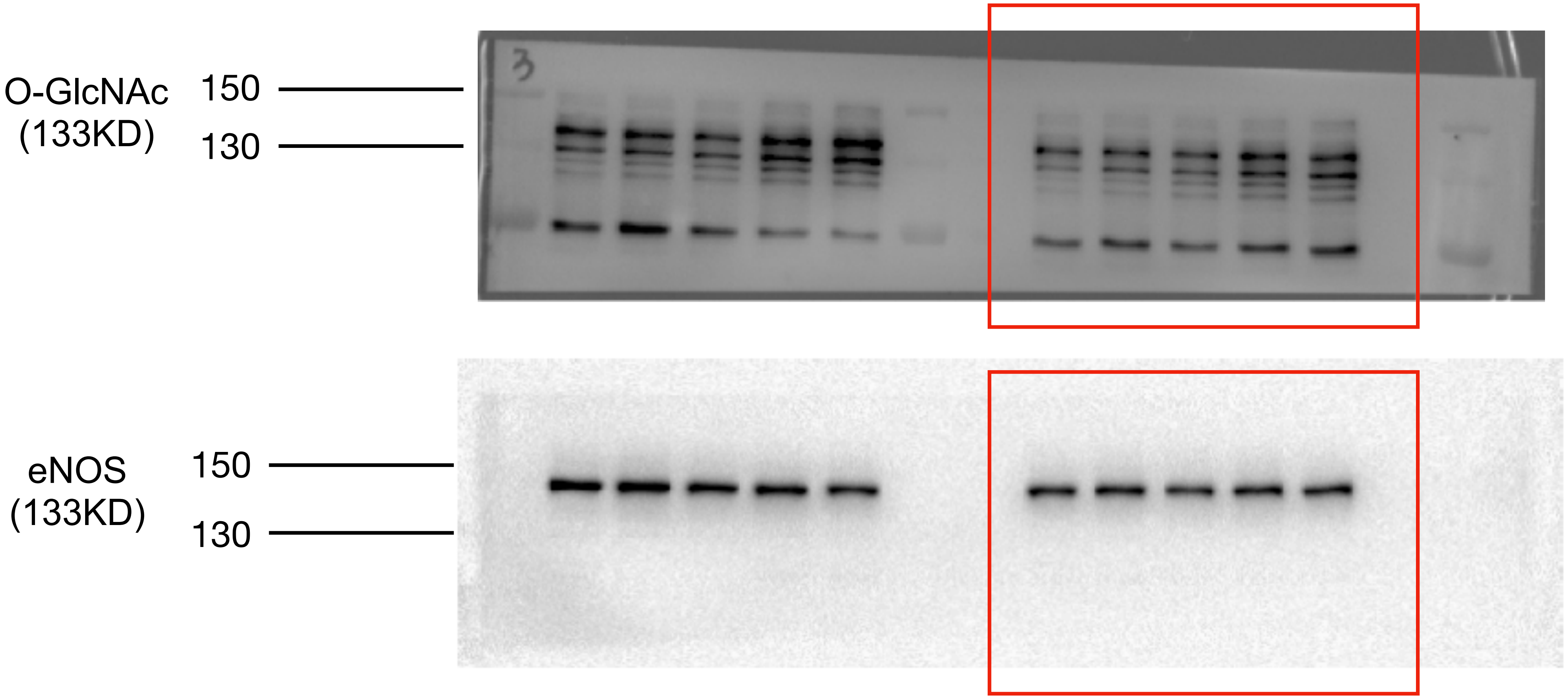

Supplementary Figure S11

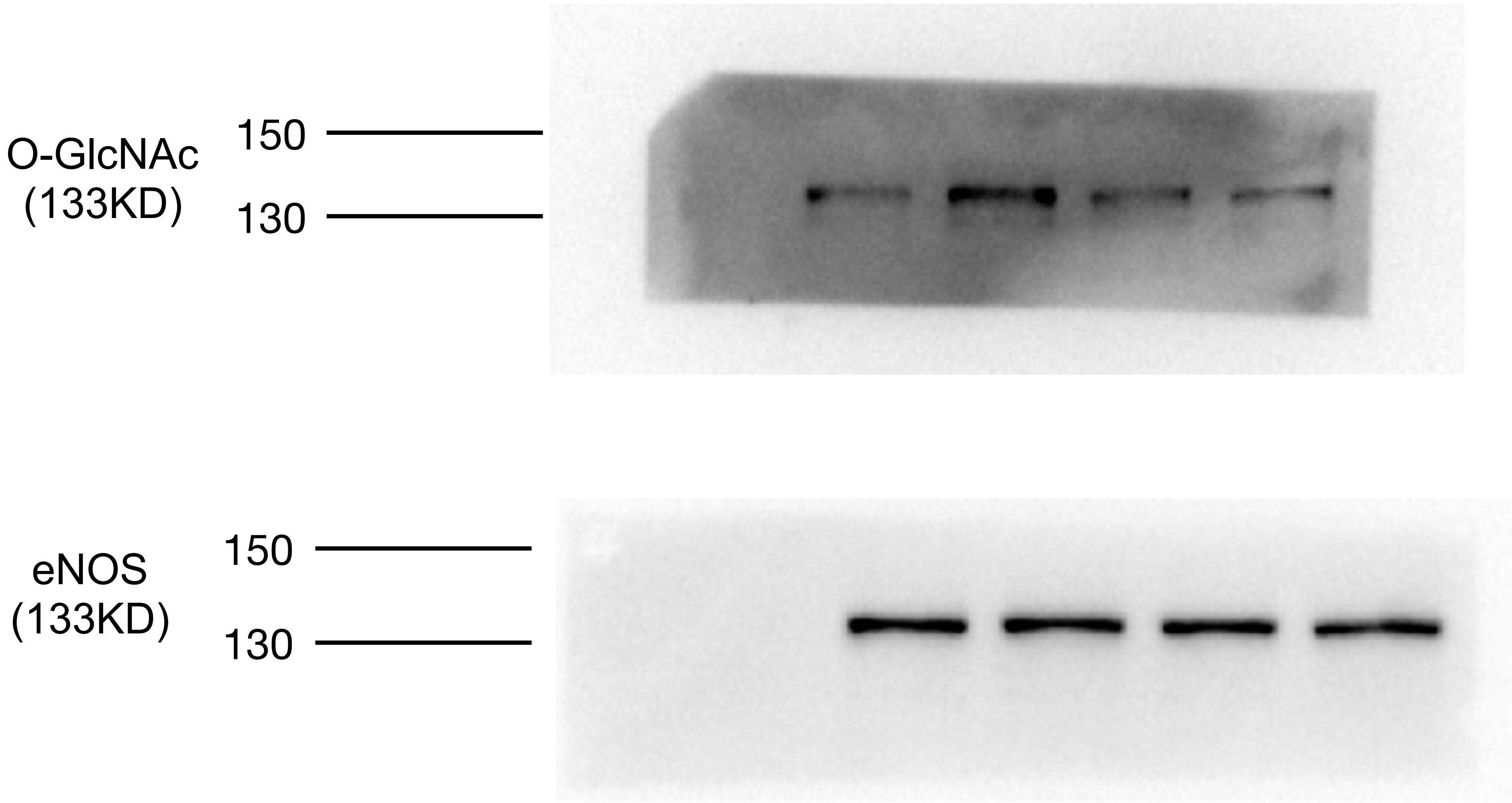

Supplementary Figure S12

The blots were cut prior to hybridisation with antibody

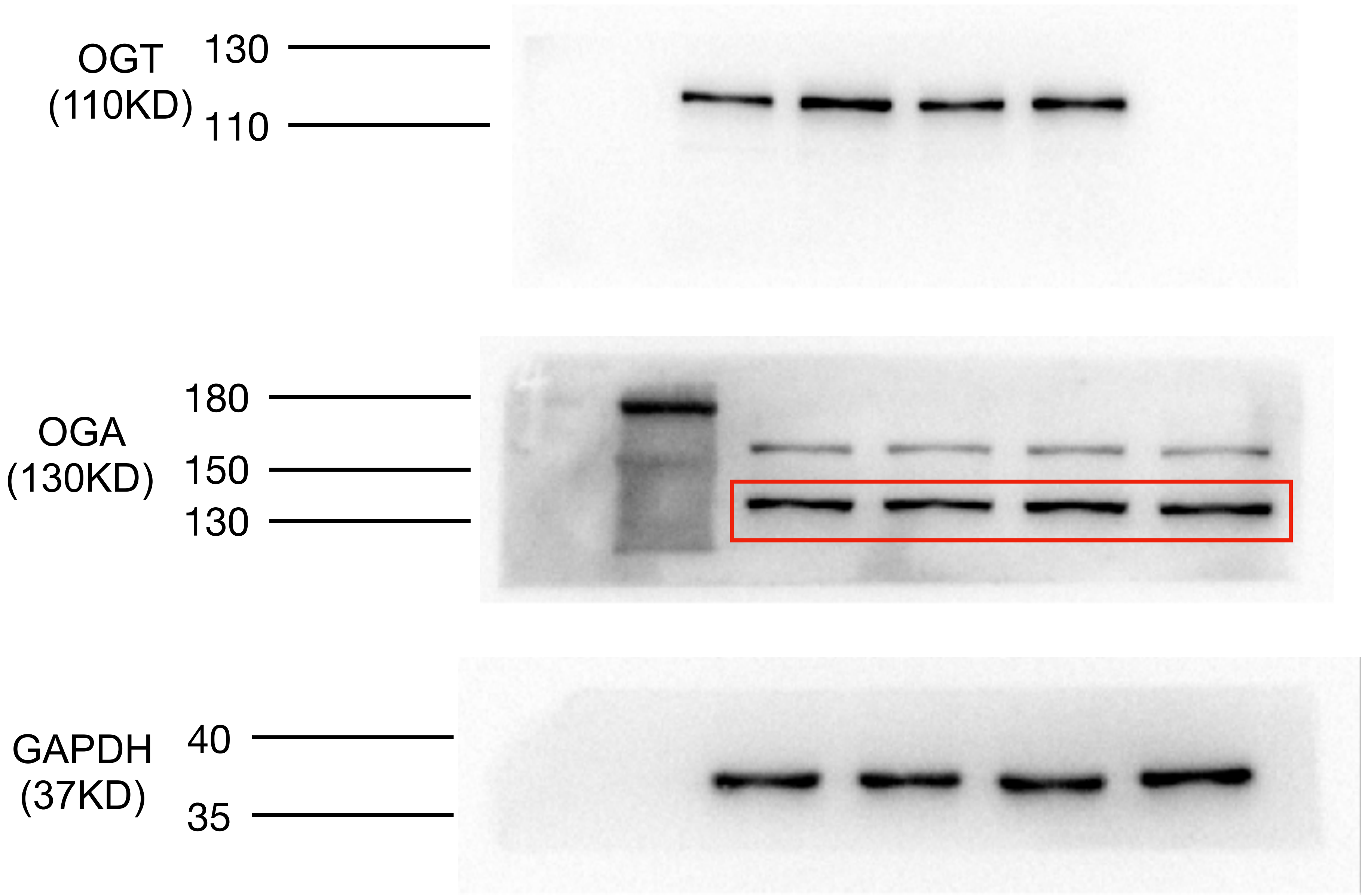

Supplementary Figure S13

OGA has a miscellaneous band. According to the molecular weight, we can determine that the down band is our target band.

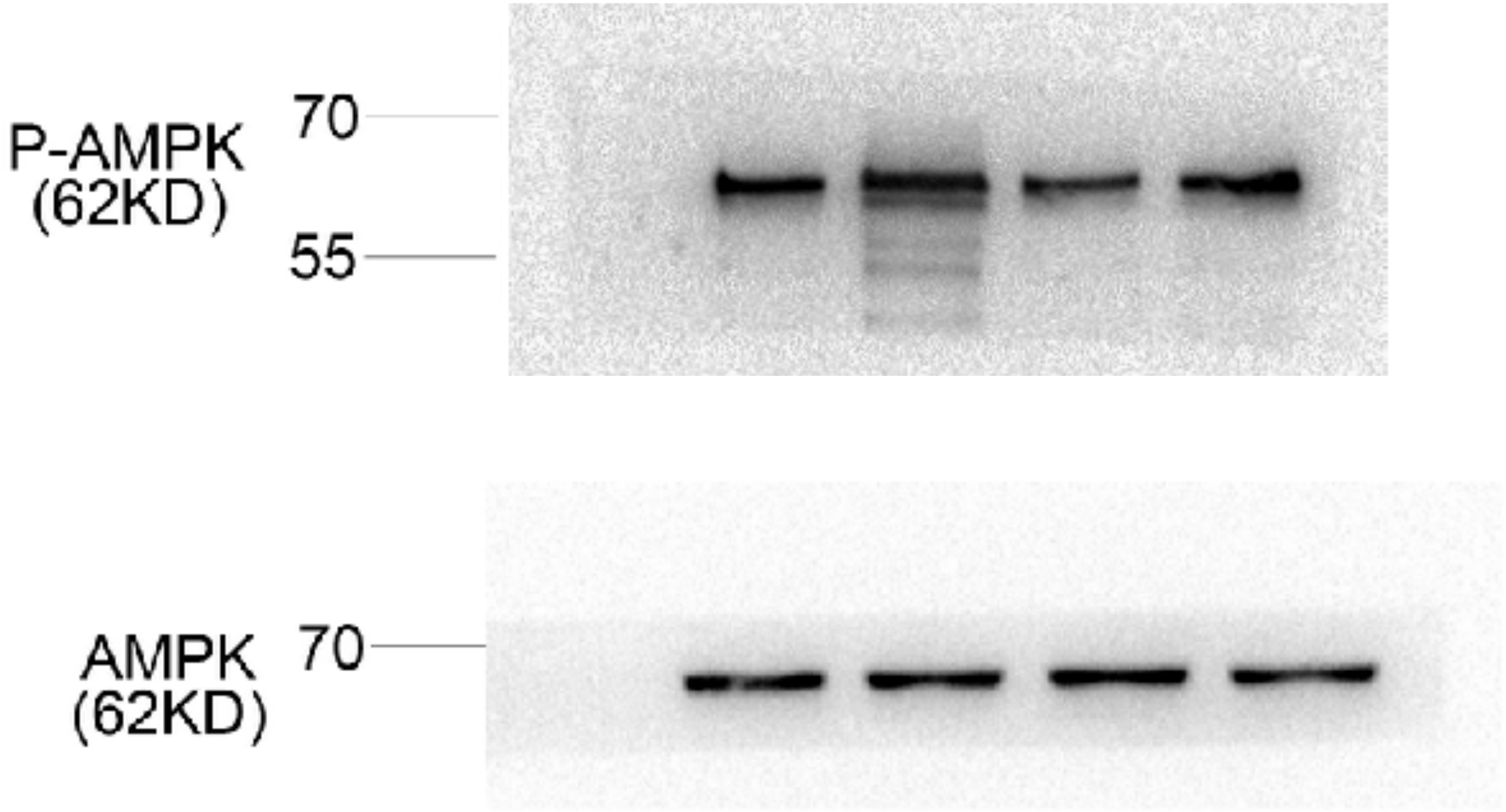

Supplementary Figure S14

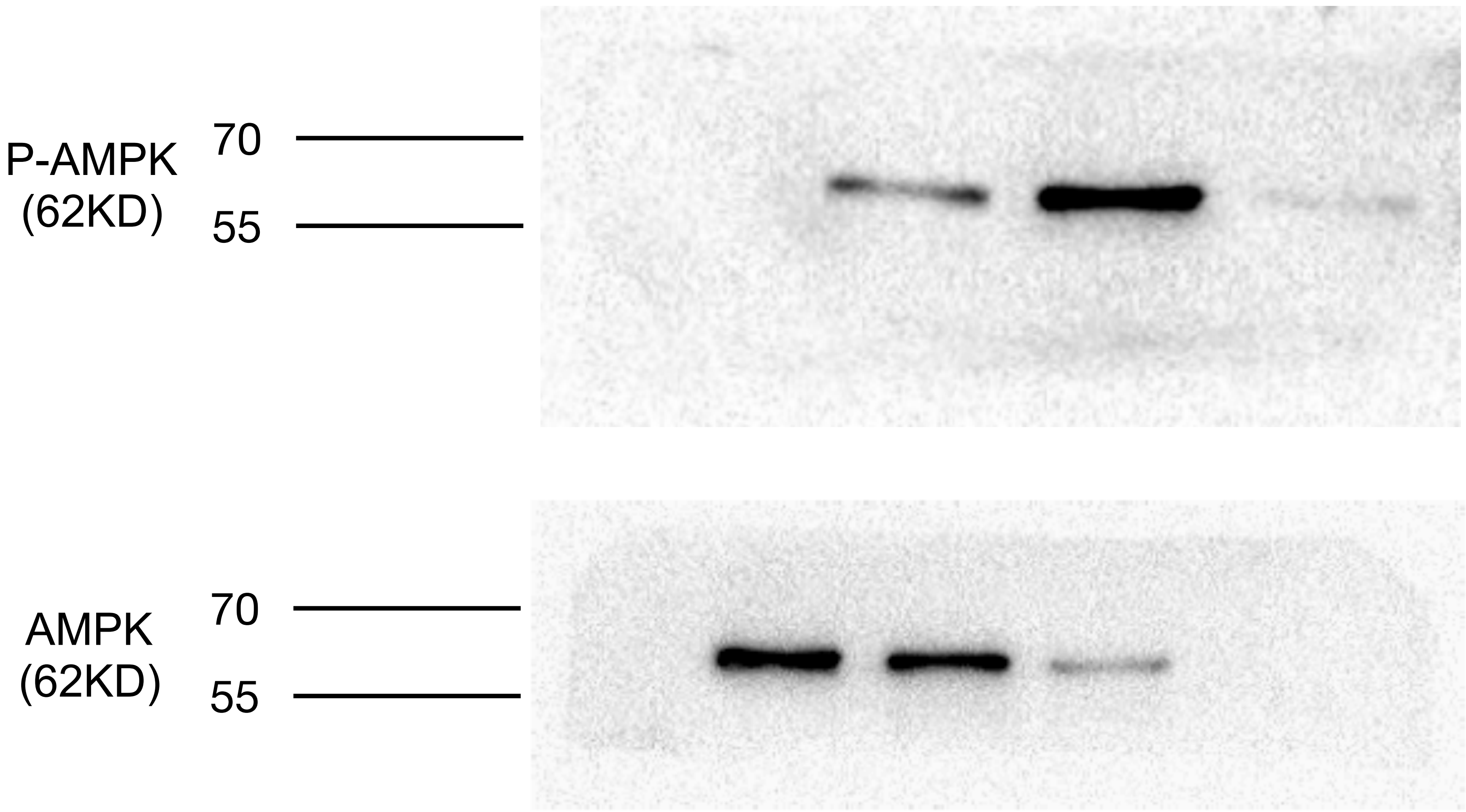

Supplementary Figure S15

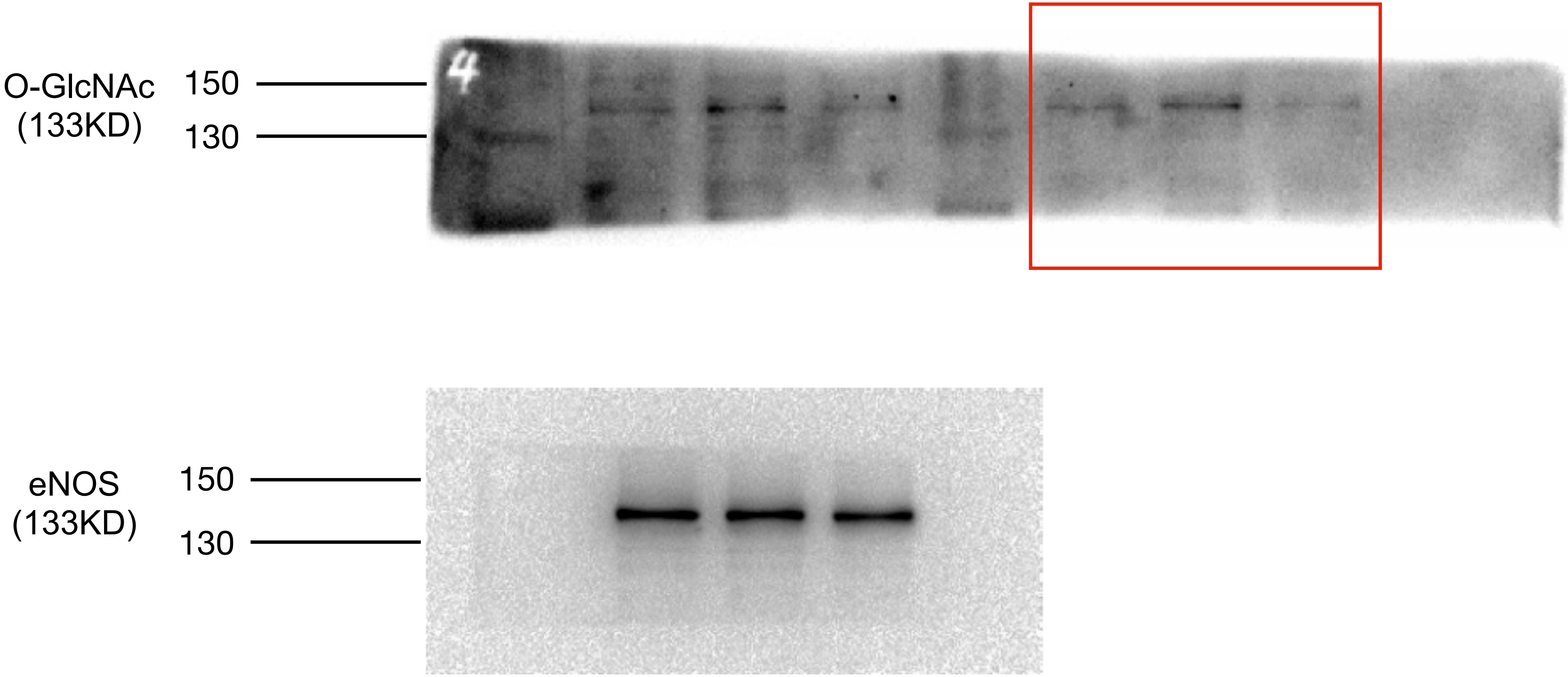

Supplementary Figure S16

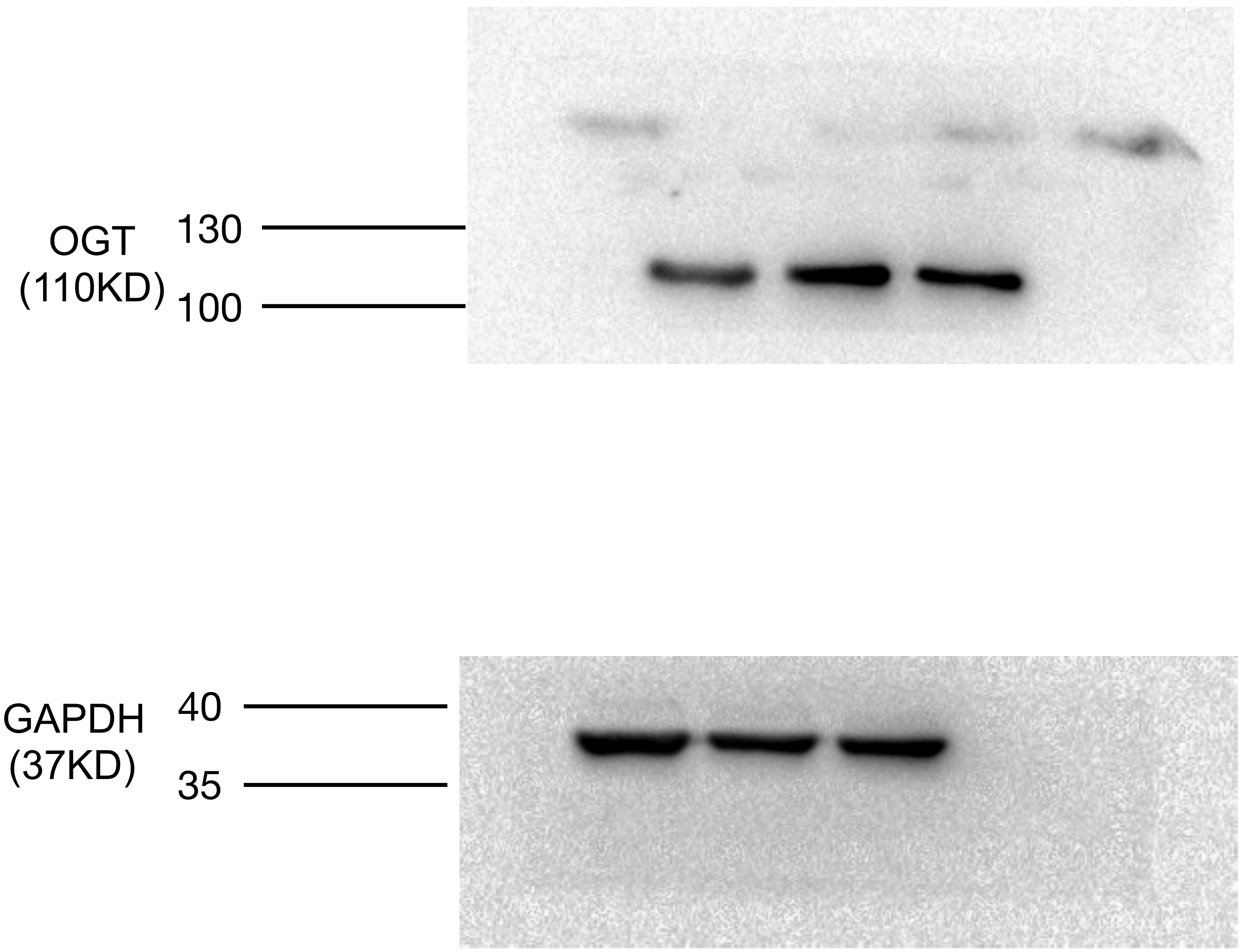

Supplementary Figure S17

The blots were cut prior to hybridisation with antibody

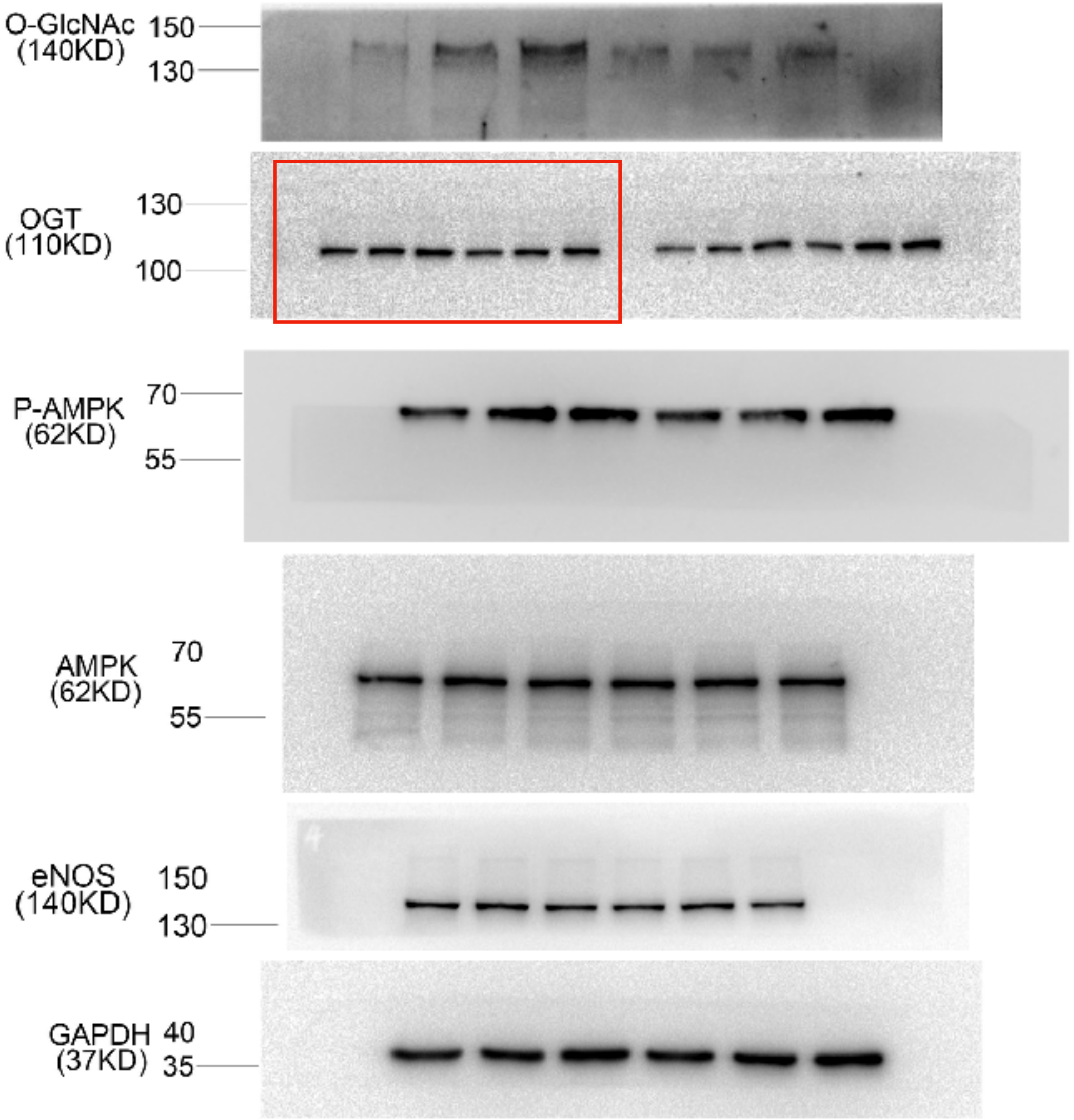

Supplementary Figure S18
